# How Antiretroviral Drug Concentrations Could Be Affected by Oxidative Stress, Physical Capacities and Genetics: A Focus on Dolutegravir Treated Male PLWH

**DOI:** 10.3390/antiox14010082

**Published:** 2025-01-13

**Authors:** Jessica Cusato, Anna Mulasso, Micol Ferrara, Alessandra Manca, Guido Accardo, Alice Palermiti, Miriam Antonucci, Gianluca Bianco, Francesco Chiara, Jacopo Mula, Maria Cristina Tettoni, Simone Cuomo, Giulia Trevisan, Stefano Bonora, Giovanni Di Perri, Corrado Lupo, Alberto Rainoldi, Antonio D’Avolio

**Affiliations:** 1 Laboratory of Clinical Pharmacology and Pharmacogenetics, Department of Medical Sciences, University of Turin, Amedeo di Savoia Hospital, 10149 Turin, Italy; jessica.cusato@unito.it (J.C.); alice.palermiti@unito.it (A.P.); jacopo.mula@unito.it (J.M.); antonio.davolio@unito.it (A.D.); 2 NeuroMuscular Function|Research Group, School of Exercise and Sport Sciences, SUISM, Department of Medical Sciences, University of Turin, 10126 Turin, Italy; anna.mulasso@unito.it (A.M.); simone.cuomo@unito.it (S.C.); corrado.lupo@unito.it (C.L.); alberto.rainoldi@unito.it (A.R.); 3 ASL Città di Torino, Amedeo di Savoia Hospital, 10149 Turin, Italy; micol.ferrara29@gmail.com (M.F.); miriam.antonucci@aslcittaditorino.it (M.A.); mariacristina.tettoni@aslcittaditorino.it (M.C.T.); 4 Unit of Infectious Diseases, Department of Medical Sciences, University of Turin, Amedeo di Savoia Hospital, 10149 Turin, Italy; guido.accardo@unito.it (G.A.); giulia.trevisan@unito.it (G.T.); stefano.bonora@unito.it (S.B.); giovanni.diperri@unito.it (G.D.P.); 5 Laboratory of Clinical Pharmacology S. Luigi A.O.U., Department of Clinical and Biological Sciences, University of Turin, Regione Gonzole, Orbassano, 10043 Turin, Italy; francesco.chiara@unito.it

**Keywords:** antioxidants, physical capacities, ROS, HAART, antiretroviral treatment

## Abstract

High levels of reactive oxygen species (ROS) are present in people living with HIV (PLWH), produced by intense physical activity; in response, our body produces antioxidant molecules. ROS influence the expression of gene-encoding enzymes and transporters involved in drug biotransformation. In addition, pharmacogenetics can influence transporter activity, and thus drug exposure. Currently, no studies concerning this topic are present in the literature. The aim of this study was to investigate whether some antioxidant molecules, physical exercise, and genetic variants could affect dolutegravir (DTG) concentrations in PLWH, switching from triple to dual therapy. Thirty PLWH were recruited and analyzed at baseline (triple therapy), and 6 months after (dual therapy). Physical capacities were investigated using validated tools. Drug concentrations and oxidative stress biomarkers levels were evaluated through liquid chromatography coupled with tandem mass spectrometry, while genetic variants through real-time PCR. No statistical differences were suggested for drug concentrations, with the exception of intracellular DTG (*p* = 0.047). Statistically significant correlations between DTG plasma concentrations and white blood cells (*p* = 0.011; S = 0.480) and cytoplasmic N-acetyl-cysteine (*p* = 0.033; S = −0.419) were observed. Finally, white blood cells and BMI remained in the final multivariate regression model as predictors of DTG concentrations. This is the first study showing possible factors related to oxidative stress impacting DTG exposure.

## 1. Introduction

The management of human immunodeficiency virus (HIV) infection has made significant advancements over the past few decades, with the development of highly active antiretroviral therapy (HAART). Among the treatments, triple and dual therapy have emerged as effective approaches in suppressing viral load and preventing the progression of the disease [1].

Triple therapy is the gold standard for HIV viral replication control and for increasing the life expectancy of people living with HIV (PLWH) [2]. These types of treatment generally include two nucleoside reverse transcriptase inhibitors (NRTI), in association with a protease inhibitor (PI), a non-nucleoside reverse transcriptase inhibitor (NNRTI), or an integrase strand transfer inhibitor (INSTI) [3,4]. Dual therapy is basically composed of an NRTI-sparing HAART or a single NRTI with another antiretroviral [5]. In particular, two of these regimens, dolutegravir (DTG) in association with lamivudine (3TC), in naïve and suppressed patients, and DTG/rilpivirine (RPV) in suppressed patients, have shown a great safety profile and efficacy [6,7,8].

Possible side effects related to long-term combination HAART can occur, requiring risk-reduction strategies [9]. In this context, monitoring drug exposure is important: therapeutic drug monitoring (TDM) is the laboratory practice that evaluates drug concentration in a specific biological matrix, basically plasma, in order to understand if drug exposure is within the therapeutic range, which is related to the high probability of efficacy and reduced risk of side effects [10].

As shown, drug concentrations could be affected by different factors, such as Reactive Oxygen Species (ROS). These molecules are implied in cell signaling and homeostasis, but they can also induce oxidative stress when produced in excess (for example, with intense physical activity [11]), leading to damage of lipids, proteins, and DNA. Furthermore, oxidative stress is involved in several pathological conditions, including inflammation, aging, and cancer [12]. Concerning HIV, high ROS levels are quantified in HIV-infected cell cultures [13,14]. Indeed, understanding the relationship between HIV, treatment, and ROS production is important for different reasons. First, oxidative stress can contribute to HIV-related comorbidities, such as cardiovascular disease or neurocognitive disorders [15,16]. Second, oxidative stress can affect drug levels and probably efficacy or toxicity [17]: in fact, ROS are able to modulate the expression of the genes encoding some drug-related transporters, such as P-glycoprotein, encoded by the *ABCB1* gene.

In order to reduce oxidative stress, our body is able to produce some antioxidant molecules, such as N-acetyl-cysteine (NAC), which is a precursor of the intracellular cysteine and glutathione (GSH). NAC is able to reduce ROS activity thanks to its scavenging property via the redox potential of thiols, or via increasing intracellular GSH concentrations [18]. Moreover, GSH is able to affect the expression of some transporters, such as ABCB1 [19,20].

Unfortunately, the link between all these variables remains to be clarified. Consequently, the aim of this study was to investigate if oxidative stress and physical capacities could influence drug concentrations in PLWH switching from triple to dual therapy, particularly focusing on DTG.

## 2. Materials and Methods

Treatment-naïve PLWH aged between 30 and 50 years were enrolled in this study. Two timings were evaluated: the first was the baseline, before starting therapy (triple therapy) and the second after six months of therapy (dual therapy). During this period enrolled PLWH maintained the same habits.

Individuals were enrolled at the Unit of Infectious Diseases at Amedeo di Savoia Hospital (Turin, Italy) from 2022 to 2023; their hematochemical tests were described.

The present study was approved by the Ethics Committee (Study Prot No. 17/2022, 16 March 2022, Comitato Etico Interaziendale Città della Salute e della Scienza, Turin, Italy). Each PLWH signed an informed consent for collecting blood samples for future analyses.

PLWH were classified into sedentary and non-sedentary individuals. Physical capacities were evaluated using (i) Finger Tapping (10 s of tapping as fast as possible for each of the hands) for manual dexterity, (ii) YMCA Step (stepping for 3 min at 96 beats/minute, heart rate recorded after 1 min of recovery) for cardiorespiratory fitness, (iii) hand-grip (4–5 s of maximum voluntary contraction, with one attempt for each hand) and Sit to Stand (5 times sit to stand, the result is the time) for strength, (iv) One Leg Stance (single leg stance maintained for a maximum of 45 s, performed on the right and left legs) for balance, and (v) Sit and Reach (sitting on the floor opposite the box, leg straight, bend forward smoothly as far as possible; the distance reached is recorded) for flexibility.

Anthropometric parameters such as weight, height, body mass index (BMI), waist circumference, and waist–hip ratio were monitored.

Antioxidant molecules were quantified both in the cytosol and in mitochondria using liquid chromatography coupled with tandem mass spectrometry.

The mitochondria isolation was performed with “Mitochondria Isolation Kit for Cultured Cells^®^” (by Thermo Scientific, Segrate, Milan, Italy). The procedure followed the Type A scheme outlined in the kit instructions. The separation protocol, in summary, included the steps reported in Supplementary Figure S1.

Considering drug exposure assessment, blood samples were collected at the conclusion of the dosing interval to obtain the trough concentration (C_trough_). Plasma was obtained using lithium heparin tubes, after centrifugation at 1400× *g* for 10 min at 4 °C. After heat inactivation, drug concentrations were quantified using a previously validated liquid chromatography–tandem mass spectrometry (LC-MS/MS) method [21].

Peripheral blood mononuclear cells (PBMCs) were isolated using cell preparation tubes (CPT^®^, Becton, Dickinson and Co., Franklin Lakes, NJ, USA, 2 CPT of 8 mL each for PBMC isolation) and centrifuged for 15 min at 1600× *g* at room temperature (25 °C).

Blood samples were processed by using a previously described protocol [22,23].

In detail, the cells were transferred into a Falcon tube using a Pasteur pipette, adjusted to a final volume of 50 mL, and rinsed twice with 0.9% sodium chloride solution. Subsequently, they were centrifuged at 2200× *g* for 6 min at 4 °C. Before the second wash, the pellet was treated with 2 mL of an ammonium salt solution (130 mM ammonium chloride + 7.5 mM ammonium carbonate) for 1 min to lyse red blood cells. The volume was then readjusted to 40 mL using 0.9% sodium chloride solution. Then, 500 μL of the cell suspension was diluted to a final volume of 19.5 mL with Isoton and divided into two portions. These aliquots were used for cell counting and mean cell volume (MCV) measurement using a Beckman Coulter Z2 analyzer (Instrumentation Laboratory, Milan, Italy). The Z2 AccuComp software (version 3.01) was used to process the results.

To prepare blank PBMC aliquots, the resulting PBMC pellet was dissolved in an extraction solution (methanol:water, 70:30 *v*/*v*). The lysates were then aliquoted and stored at −80 °C.

Genetic variants were evaluated through real-time PCR (CFX 96, Biorad, Milan, Italy).

ANOVA tests were used to treat the data. The Shapiro–Wilk test was used for normality evaluation for all the considered variables. Non-normal variables were resumed as median values and interquartile range (IQR), while dichotomy variables as numbers and percentages.

Kruskal–Wallis and Mann-Whitney were used to test differences between linear and dichotomic variables, such as antioxidant molecules according to sedentary or not people.

Tests were performed with IBM SPSS Statistics 28.0 for Windows (Chicago, IL, USA).

## 3. Results

### 3.1. PLWH Characteristics

In this study, 30 PLWH were recruited, but 5 individuals were not present at the follow-up. Furthermore, considering physical measures, data were available only for 22 subjects. PLWH characteristics were reported in Table 1, whereas administered drugs are resumed in Table 2. The median age was 42.5 years (IQR 35.8–48) and the median body mass index (BMI) was 23.3 Kg/m^2^ (IQR 22.2; 24.9). All enrolled individuals were male.

Oxidative stress-related molecular data are reported in Supplementary Table S1.

### 3.2. Drug Plasma and Intracellular Exposure

**Figure 1:**
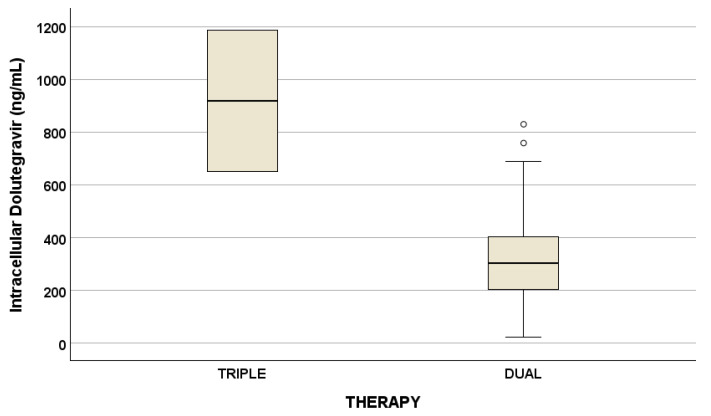
Plasma and intracellular drug concentrations at the two different timings (*p* = 0.047, ng/mL), described as median concentration and interquartile ranges. Outliers are represented by little circles.

Drug concentrations are reported in Table 3. No statistical differences were suggested for drug concentrations considering triple and dual therapy, with the exception of intracellular DTG (Figure 1).

### 3.3. Hematochemical Values, Cytoplasm/Mitochondrial Redox Factors, and Physical Capacities Role in Affecting DTG Concentrations

Since most PLWH are treated with DTG both in triple or dual regimens and a difference between triple and dual therapy in terms of intracellular DTG concentrations, we decided to focus on DTG evaluation.

**Figure 2:**
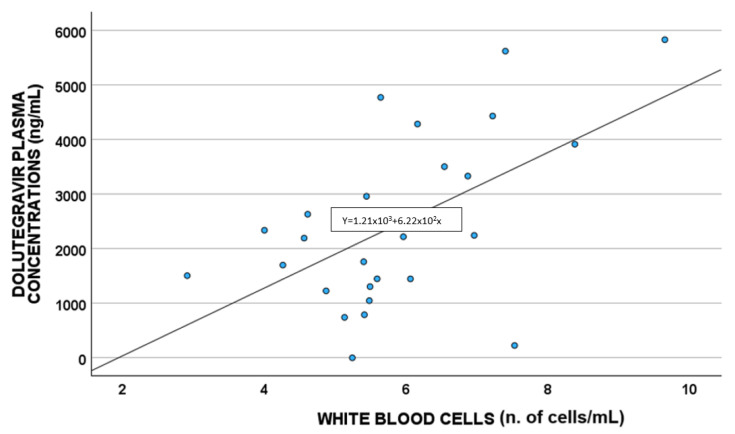
Correlations between dolutegravir plasma exposure and white blood cells (n. of cells/mL).

**Figure 3:**
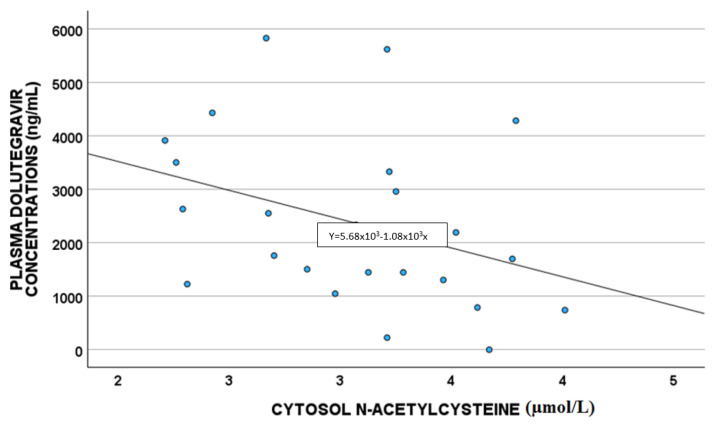
Correlations between dolutegravir plasma exposure and cytoplasm NAC (µmol/L).

A statistically significant correlation between DTG plasma concentrations and white blood cells (*p* = 0.011; S = 0.480) and cytoplasm NAC (µmol/L) (*p* = 0.033 S = −0.419) was highlighted, as shown in Figure 2 and Figure 3, respectively.

No correlations between plasma DTG and physical capacities were observed; in particular, all the physical tests evaluated in this study are not correlated with DTG concentrations for both triple and dual therapy.

In addition, correlations between ROS levels in mitochondria with NAC levels were performed, suggesting only mitochondrial taurine correlation with NAC cytosol levels.

### 3.4. Genetics Impact

No genetic variant was observed to impact DTG drug concentrations, both in triple and dual therapy.

### 3.5. Regression Analysis

**Figure 4:**
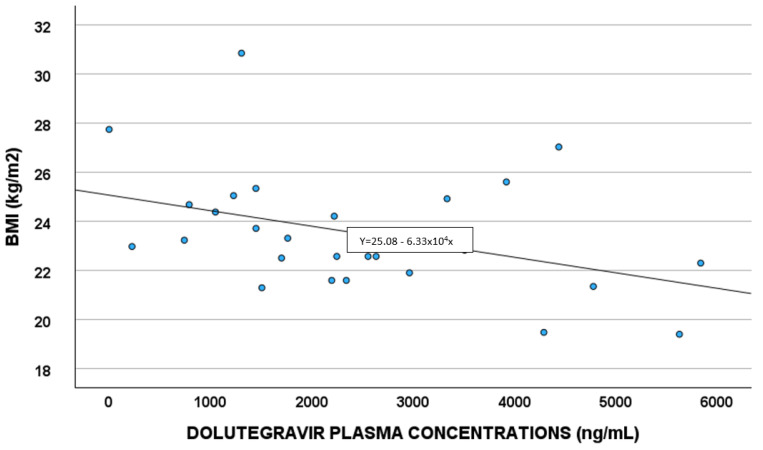
Correlations between dolutegravir plasma exposure and body mass index (BMI).

Demographic, pharmacological, and genetic factors able to predict DTG concentrations were evaluated in the linear regression analysis (Table 4). White blood cells and BMI (Figure 4) remained in the final multivariate model.

## 4. Discussion

DTG is a second-generation INSTI with a 90% inhibitory concentration (IC90) of 0.064 μg/mL for wild-type virus in vitro. It has a half-life of 13–14 h and maintains effective concentrations above IC90 for over 30 h after a single dose. DTG shows low interindividual variability, and its efficacy is closely tied to its trough level (C_trough_). It is also effective against INSTI-resistant phenotypes.

In this study, a total of 620 DTG plasma concentrations were quantified in 521 PLWH [24] describing DTG pharmacokinetics as a one-compartment model with first-order absorption and elimination. Basically, the DTG apparent volume of distribution was 20.2 L, its apparent clearance was 0.93 L/h with 32% between-individual variability and the absorption rate constant was 2.24–1 h. For the elderly, increased body weight and smoking were related to higher clearance. Furthermore, atazanavir co-administration reduces DTG clearance by 38%, while darunavir slightly increases clearance by 14%. The higher impact on DTG clearance is shown by rifampicin co-administration: in fact, simulations highlighted that the median DTG trough levels are 63% lower after administration with 50 mg/12 h of rifampicin compared to the standard dosage of 50 mg/24 h without rifampicin.

DTG is generally well tolerated. It inhibits the renal and neuronal transporter organic cation transporter 2 (OCT2), leading to decreased tubular secretion of creatinine, and thus a consequent increase in serum creatinine, not associated with decreased glomerular filtration rate or progressive renal impairment (it is a “cosmetic effect”). DTG is mainly metabolized by UDP glucuronosyltransferase (UGT)1A1 and cytochrome (CYP) 3A4 and it neither induces nor inhibits CYP-450 isozymes (with a consequent reduced risk of interaction). However, antiacid drugs significantly reduce DTG plasma concentrations and should be administered 2 h before or 6 h after DTG dose.

DTG is a substrate of both ABCB1 and ABCG2 transporters, whose activity could be influenced by ROS [25]. In fact, ROS can downregulate P-glycoprotein (encoded by *ABCB1* gene expression). Thus, this could influence drug exposure, and, probably, the efficacy or tolerability. High ROS levels are quantified in HIV-infected cell cultures [13,14]. In fact, PLWH showed reduced antioxidant activity, low GSH/GSSG ratio, and low GSH concentrations in blood [26]. In our recent article, we suggested a statistically significant difference between triple and dual therapy in terms of mitochondrial GSH; in particular, it is reduced in triple therapy compared to dual therapy [27].

Currently, no studies evaluating drug exposure (particularly for DTG) according to antioxidant molecules and physical capacities are present in the literature. Consequently, in this work, we evaluated plasma and intracellular drug concentrations comparing triple and dual therapy: only intracellular DTG was different between the two therapies with a *p*-value of 0.047. Particularly, intracellular DTG concentrations are reduced in dual therapy compared to triple therapy (but considering that only two PLWH were administered DTG in triple therapy).

Plasma DTG concentrations were correlated with cytoplasm NAC and the number of white blood cells [28].

NAC has anti-inflammatory and antioxidant activity, and it is described as able to reverse the damaging effects of HAART in vitro and in vivo; in particular, in rats, NAC treatment mitigated the toxic effect of therapy. As suggested before, ABCB1 expression is up-regulated by ROS levels and GSH depletion [19,20,29]; NAC is able to reverse this mechanism, leading to ROS reduction, and thus ABCB1 down-regulation. Thus, in the case of high NAC levels, we were supposed to have increased DTG concentrations, whereas in our study, we found low drug levels, without confirming the literature. Probably, DTG reduced concentrations could be related to other factors linked to the detoxification directly performed by GSH (e.g., glutathione transferase activity); in fact, higher NAC levels are associated with higher GSH concentrations, which could detoxify the cells from drugs, such as DTG, which consequently is present in lower levels, as reported in our work [30,31].

Concerning white blood cells, a positive correlation between these cells and DTG concentrations was suggested, also confirmed in the linear regression multivariate analysis; this agrees with that reported by Echefu et al., who found a higher total white cell count in PLWH administered DTG compared to those treated with ritonavir [28].

A correlation between DTG plasma exposure and BMI was observed; a study by Berton et al. [32] showed obesity as having a slight impact on DTG concentrations, when considering a BMI of 30–42 Kg/m^2^. In our work, the correlation seems to be present also at lower levels of BMI.

No VD level reductions upon transition from triple to double therapy were suggested in this work: this was probably related to most of the blood being withdrawn during winter, when VD levels are reduced, for dual therapy, and during summer for triple therapy, when VD levels are increased [33]. In this study, no correlations between plasma DTG and physical capacities were observed, probably due to the small number of analyzed patients or because DTG biotransformation and transport-related enzymes and transporters expression is not influenced by physical exercises, as reported for other proteins in other contexts, as reported by Plaza-Diaz in diabetes, for example [34].

Moreover, no impact of genetics on DTG plasma exposure was observed in the regression analysis; this could be due to the low number of PLWH. In fact, one of the limitations of this study is the small number of enrolled PLWH, but the cost of analyzing all the antioxidant molecules was high. In addition, a single cohort was analyzed.

## 5. Conclusions

In conclusion, this study is the first to investigate the differences in terms of oxidative stress and physical capacities in PLWH switching from triple to dual therapy. In particular, no statistical differences were suggested for drug concentrations considering triple and dual therapy, with the exception of intracellular DTG; white blood cells and BMI, but not oxidative stress and pharmacogenetic-related factors, remained in the final multivariate regression model as predictors of DTG concentrations.

In the future, it would be interesting to evaluate the association between antiretroviral drug concentrations, antioxidant molecules, physical capacities, and genetics in larger and different cohorts of PLWH, other than focusing on women and the effect of the hormonal stage.

## Figures and Tables

**Table 1:** Characteristics of the population. WBC = white blood cells; RBC = red blood cell; HBG = Hemoglobin; HCT = hematocrit test; PLT = platelet count; HDL = high-density lipoproteins; LDL = low-density lipoprotein; AST = Aspartate transferase; ALT = alanine transaminase; GGT = gamma-glutamyl transferase; LDH = lactate dehydrogenase; CK = creatine kinase; IQR = Interquartile range.

Variables, Units (Reference Intervals)	Triple Therapy		Double Therapy		
	MEDIAN	IQR	MEDIAN	IQR	*p*-Value
**WEIGHT, Kg**	71.00	65.75; 80.00	70.00	65.75; 87.75	
**WBC, % (4–10)**	5.69	4.72; 7.30	5.54	5.16; 6.45	0.865
**RBC, 10^12^/L (4–5)**	4.86	4.68; 5.12	4.84	4.57; 5.06	0.985
**HGB, g/L (120–160)**	153.50	144.30; 158.0	151.50	140.3; 157.50	0.690
**HCT, L/L (0.35–0.45)**	0.46	0.44; 0.47	0.45	0.42; 0.48	0.703
**PLT, 10^9^/L (130–390)**	265.00	215.00; 301.00	239.00	192.5; 289.3	0.478
**Total Linfocytes T% (60–87)**	76.56	69.95; 83.10	75.40	67.4; 80.7	0.413
**Helper/inducer Linfocytes % (32–61)**	36.00	32.00; 43.00	35.50	31.50; 40.00	0.674
**Suppressor/cytotoxic Linfocytes% (14–43)**	31.85	28.05; 43.00	31.30	28.70; 40.00	0.785
**CD4/CD8, % (0.9–2.5)**	1.20	0.80; 1.55	1.10	0.90; 1.38	0.802
**Glucose, mg/dL (75–110)**	84.00	78.00; 86.0	80.0	73.50; 87.80	0.634
**Creatinine, mg/dL (0.5–0.96)**	1.00	0.89; 1.09	1.01	0.90; 1.14	0.521
**Total Cholesterol, mg/dL (140–200)**	189.50	153.0; 203.80	179.00	164.00; 203.00	0.869
**HDL, mg/dL (>65)**	47.00	40.00; 62.00	51.00	41.30; 63.80	0.938
**LDL, mg/dL (<130)**	118.00	91.00; 130.00	107.50	100.80; 137.50	0.938
**Triglycerides, mg/dL (<200)**	108.50	79.30; 131.80	91	62.30; 120.30	0.285
**AST, Ul/L (5–32)**	25.50	22.80; 30.80	27.50	23.80; 31.00	0.330
**ALT, Ul/L (5–33)**	27.50	20.50; 33.80	28.00	20.00; 34.00	0.553
**GGT, Ul/L (6–42)**	19.00	16.00; 27.00	19.00	14.00; 24.00	0.861
**Alkaline phosphatase, U/L (35–104)**	62.00	52.00; 82.00	63.50	56.80; 73.80	0.938
**LDH, U/L (200–480)**	177.50	161.80; 200.80	180.50	159.30; 208.8	0.823
**CK, U/L (7–167)**	134.50	89.80; 192.50	159.50	96.00; 220.8	0.409
**Total bilirubin, mg/dL (0.1–1.2)**	0.49	0.38; 0.67	0.46	0.39; 0.59	0.726
**Sodium, mmol/L (136–145)**	141.00	140.00; 142.00	141.00	139.00; 142.00	0.525
**Potassium, mEq/L (3.5–5.1)**	4.20	4.07; 4.53	4.30	4.20; 4.50	0.399
**Calcium, mg/dL (2.1–2.5)**	2.40	2.30; 2.41	2.30	2.20; 2.40	0.105
**Phosphorus, mg/dL (2.5–4.8)**	3.05	2.80; 3.50	3.10	2.80; 3.43	0.930
**Vitamin D, ng/mL (>30)**	27.80	22.20; 36.6	21.85	17.58; 29.20	**0.026**
**HIV-RNA, copies/mL**	Not detectable		Not detectable		**-**
**dominant tapping test**	58	52–63	59	55–63	0.355
**non-dominant tapping test**	54	51–58	54	48–59	0.778
**tapping test percentile**	81	49–95	81	43–95	0.607
**dominant handgrip**	44	34–46	46	43–49	0.101
**non-dominant handgrip**	38	32–45	41	37–48	0.084
**handgrip percentile**	31.5	10.3–46.3	37.5	30.0–62.5	0.121
**sit and reach**	29	18–34	28	18–33	0.712
**sit and reach percentile**	81	33–92	73	25–93	0.938
**sit to stand**	6.14	5.38–6.90	5.65	4.42–6.06	0.103
**sit to stand percentile**	69	55–80	82	63–90	0.145
**step test**	104	88–119	96	80–114	0.277
**step test percentile**	52	26–71	60	35–80	0.242

**Table 2:** Administered drug regimens. DTG = dolutegravir; ABV = abacavir; 3TC = lamivudine; BIC = bictegravir; TAF = tenofovir alafenamide; FTC = emtricitabine; RPV = rilpivirine; DRV = darunavir; c = cobicistat.

Drugs	Triple Therapy
DTG/ABV/3TC	1 (4%)
BIC/TAF/FTC	12 (48%)
DTG/TAF/FTC	1 (4%)
RPV/TAF/FTC	10 (40%)
DRV/c/TAF/FTC	1 (4%)
	**Dual Therapy**
TG/3TC	20 (80%)
DTG/RPV	5 (20%)

**Table 3:** Plasma (p) and intracellular (i) drug concentrations at different timings (ng/mL). FTC = emtricitabine; TAF = tenofovir alafenamide; BIC = bictegravir; TFV = tenofovir; RPV = rilpivirine; DTG = dolutegravir; 3TC = lamivudine; IQR = Interquartile range.

Drugs, Units (Reference Intervals)	Triple Therapy		Dual Therapy		
	Median	IQR	Median	IQR	*p*-Value
**p-FTC**, ng/mL	44.5	34.75; 129			
**i-FTC MONOPHOSPHATE**, ng/mL	956.23	599.37; 1188.79			
**i-FTC DIPHOSPHATE**, ng/mL	12,008	8109; 14,666			
**i-FTC TRIPHOSPHATE**, ng/mL	6367	4356; 8733			
**p-TAF**, ng/mL (9.8–20.4)	n.d.	n.d.			
**p-BIC**, ng/mL (> 162)	4175	2806; 6704			
**i-BIC**, ng/mL	436.1	146.9; 631.2			
**p-TFV**, ng/mL (40–180)	17	12.25; 21			
**i-TFV**, ng/mL	364.40	259.13; 439.88			
**p-RPV**, ng/mL (50–500)	129.5	93.5; 168.5	126	72; 229	0.806
**i-RPV**, ng/mL	402.6	243.9; 492.8	282.9	214.9; 865.7	0.828
**p-DTG**, ng/mL (300–2138)	2487	2340;/	2196	1267; 3713	0.579
**i-DTG**, ng/mL	260	1026;/	302.40	200.07; 425.37	**0.047**
**p-3TC**, ng/mL	-	-	118	62.5; 177.5	

**Table 4:** Logistic regression analysis for predictors of dolutegravir plasma concentrations. BMI: body mass index. NAC: N-acetyl-cysteine.

	Dolutegravir Plasma Concentrations			
	Univariate		Multivariate	
	*p*-VALUE	OR (95% IC)	*p*-VALUE	OR (95% IC)
BMI at baseline, Kg/m^2^	**0.037**	**−257.422 (−497.763; 17.081)**	**0.005**	**−284.026 (−473.148; −94.905)**
Age, years	0.541	−24.783 (−107.157; 57.592)		
Co-medications	0.163	−1209.667 (−2943.905; 524.572)		
Dual/Triple therapy	0.971	−42.480 (−2466.657; 2381.697)		
*ABCB1 3435 TT*	0.749	−258.900 (−1914.979; 1397.179)		
*ABCB1 1236 TT*	0.156	1053.447 (−433.243; 2540.137)		
*ABCG2 421 CA/AA*	0.613	−285.005 (−1434.917; 864.907)		
*ABCG2 1194 + 328 CC*	0.090	1641.909 (−274.535; 3558.353)		
*SLC22A2 CC*	0.346	−535.400 (−1686.139; 615.339)		
*HNF4 975 GG*	0.903	−107.583 (−1918.022; 1702.855)		
*CAR TT*	0.789	266.530 (−1773.354; 2306.414)		
*PXR 63,396 CT/TT*	0.985	−14.850 (−1674.677; 1644.977)		
White blood cells, n/mL	**0.003**	**621.541 (239.289; 1003.794)**	**<0.001**	**656.828 (325.609; 988.047)**
NAC Cytosol, µmol/L	0.068	−1078.332 (−2243.935; 87.271)		
Sedentary behavior	0.805	159.088 (−1154.006; 1472.183)		

## Data Availability

Data are contained within the article.

## Supplementary Materials

The following supporting information can be downloaded at: https://www.mdpi.com/article/10.3390/antiox14010082/s1.
