# Supplementary material for: How Antiretroviral Drug Concentrations Could Be Affected by Oxidative Stress, Physical Capacities and Genetics: A Focus on Dolutegravir Treated Male PLWH
Source: Antioxidants (Basel). 2025 Jan 13;14(1):82. doi: 10.3390/antiox14010082 (PMC11759814; doi:10.3390/antiox14010082)
Supplement: Supplementary file 1 [file antioxidants-14-00082-s001.zip › antioxidants-3256502-supplementary.pdf]

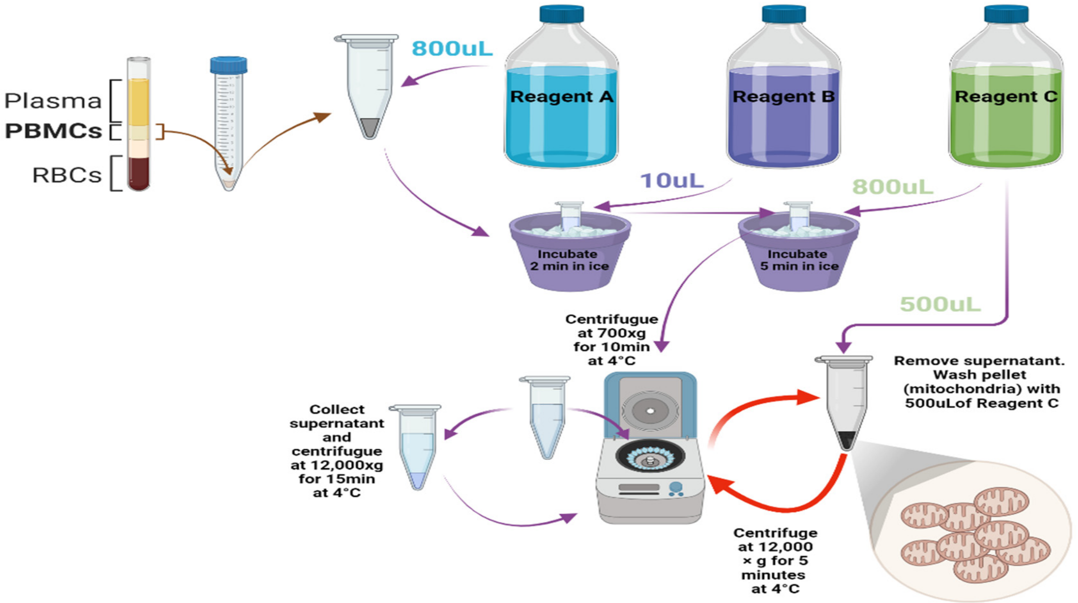

Supplementary Figure S1. Mitochondria isolation protocol.

Table S1. Quantification of antioxidant molecules in triple and dual therapies.

	**Triple Therapy**		**Double Therapy**		
	**MEDIAN**	**IQR**	**MEDIAN**	**IQR**	***p*-value**
mitochondrial cysteine	5.6	4.4 - 8.7	5.4	4.8 - 6.1	0.719
mitochondrial glycine	25.03	17.8 - 35.6	26.1	18.5 - 33.6	0.719
mitochondrial glutammic acid	11.2	9.4 - 13.1	11.1	9.9 - 13.3	0.379
mitochondrial disolphorous glutathion	0.75	0.69 - 0.78	0.75	0.65 - 0.81	0.764
mitochondrial glutathion	3.5	3.4 - 3.6	3.7	3.6 - 3.9	**0.003**
mitochondrial omocisteine	1.2	1.1 - 1.6	1.2	1.1 - 1.5	0.826
mitochondrial methyonine	2.2	1.6 - 3.0	2.3	1.9 - 3.0	0.976
mitochondrial n- acetil cysteine	1.5	1.4 - 1.6	1.6	1.2 - 1.7	0.478
mitochondrial n- formyl- methyonine	4.5	4.1 - 4.9	4.5	4.1 - 5.0	0.569
mitochondrial pyruvic acid	12.3	10.7 - 15.8	12.1	11.1 - 15.3	0.976
mitochondrial serine	2.0	1.7 - 2.4	2.0	1.7 - 2.4	0.904
mitochondrial taurine	2.0	1.1 - 2.2	1.9	0.9 - 2.2	0.881
mitochondrial s- adenosil methyonine	0.11	0.08 - 0.13	0.12	0.10 - 0.13	0.207
mitochondrial s- adenosil omocisteyne	0.0053	0.0039 - 0.0073	0.0055	0.0041 - 0.0066	0.849
cytosol cysteine	3.9	3.6 - 4.8	4.2	3.8 - 4.5	0.285
cytosol glycine	7.1	6.3 - 8.3	7.4	6.9 - 8.2	0.308
cytosol glutammic acid	8.4	4.1 - 10.0	6.9	5.5 - 9.1	0.646
cytosol disolphorous glutathion	0.62	0.52 - 0.66	0.61	0.54 - 0.65	0.795

cytosol glutathion	35.0	25.3 - 56.1	33.6	25.8 - 57.6	0.834
cytosol omocisteine	1.8	0.8 - 4.5	1.2	0.9 - 4.6	0.772
cytosol methyonine	2.9	2.1 - 3.2	3.1	2.6 - 3.7	0.267
cytosol n- acetil cysteine	3.2	2.9 - 3.6	3.2	2.7 - 3.5	0.810
cytosol n- formyl- methyonine	6.2	5.3 - 7.4	6.3	5.5 - 7.0	0.582
cytosol pyruvic acid	15.6	12.6 - 17.4	14.5	12.9 -17.0	0.976
cytosol serine	3.0	2.1 - 3.2	2.7	1.9 - 3.3	0.490
cytosol taurine	16.5	14.0 - 29.0	15.8	14.1 - 17.7	0.298
cytosol s- adenosil methyonine	0.18	0.15 - 0.40	0.16	0.13 - 0.22	0.193
cytosol s- adenosil omocisteyne	0.0280	0.0135 - 0.0570	0.0284	0.0171 - 0.0387	0.944
